# The Use of High-Density SNP Array to Map Homozygosity in Consanguineous Families to Efficiently Identify Candidate Genes: Application to Woodhouse-Sakati Syndrome

**DOI:** 10.1155/2015/169482

**Published:** 2015-11-17

**Authors:** Molly B. Sheridan, Elizabeth Wohler, Denise A. S. Batista, Carolyn Applegate, Julie Hoover-Fong

**Affiliations:** ^1^McKusick-Nathans Institute of Genetic Medicine, Johns Hopkins University School of Medicine, Baltimore, MD 21287, USA; ^2^Cytogenomics Laboratory, Johns Hopkins Hospital, Baltimore, MD 21287, USA; ^3^Department of Pathology, Johns Hopkins University School of Medicine, Baltimore, MD 21287, USA

## Abstract

Two consanguineous Qatari siblings presented for evaluation: a 17-4/12-year-old male with hypogonadotropic hypogonadism, alopecia, intellectual disability, and microcephaly and his 19-year-old sister with primary amenorrhea, alopecia, and normal cognition. Both required hormone treatment to produce secondary sex characteristics and pubertal development beyond Tanner 1. SNP array analysis of both probands was performed to detect shared regions of homozygosity which may harbor homozygous mutations in a gene causing their common features of abnormal pubertal development, alopecia, and variable cognitive delay. Our patients shared multiple homozygous genomic regions; ten shared regions were >1 Mb in length and constituted 0.99% of the genome.* DCAF17*, encoding a transmembrane nuclear protein of uncertain function, was the only gene identified in a homozygous region known to cause hypogonadotropic hypogonadism.* DCAF17* mutations are associated with Woodhouse-Sakati syndrome, a rare disorder characterized by alopecia, hypogonadotropic hypogonadism, sensorineural hearing loss, diabetes mellitus, and extrapyramidal movements. Sequencing of the coding exons and flanking intronic regions of* DCAF17* in the proband revealed homozygosity for a previously described founder mutation (c.436delC). Targeted* DCAF17* sequencing of his affected sibling revealed the same homozygous mutation. This family illustrates the utility of SNP array testing in consanguineous families to efficiently and inexpensively identify regions of genomic homozygosity in which genetic candidates for recessive conditions can be identified.

## 1. Introduction

Woodhouse-Sakati syndrome (WSS, MIM 241080) is a rare, multisystem autosomal recessive disorder that was first described in two consanguineous families from Saudi Arabia in 1983 [1]. To date, approximately 84 WSS patients in 29 families have been reported in the literature [2–16]. While WSS has been identified predominantly in patients of Middle Eastern origin, it has been described in four European families (Italy, France, and Eastern Europe) and two patients from South Asia (South India and Pakistan) [2, 3, 5–10].

The WSS phenotype is variable within and among families but is characterized overall by alopecia, hypogonadotropic hypogonadism (HH), sensorineural hearing loss, diabetes mellitus, and extrapyramidal movements. The alopecia is often present in early childhood in affected individuals, potentially involving scalp hair, eyebrows, eyelashes, and pubic and axillary hair [15]. HH becomes apparent when pubertal development is delayed or fails to occur, thus a later sign of WSS [16]. Exogenous hormone therapy can promote secondary sex characteristic development, as in our male patient presented here. Streak, hypoplastic, and absent gonads and uterine structures have also been described in WSS [5, 7, 10, 11, 15, 16]. Hearing loss may be mild to profound though not present in all affected individuals. The diabetes which develops in the majority of WSS patients may require insulin for adequate glucose control though insulin resistance is not common. The extrapyramidal features of WSS are varied, ranging from focal dystonia and chorea which evolves to be generalized. Several published reports of the movement disorder associated with WSS indicate that it begins in the 2nd decade or beyond but is not identified in all patients with WSS [4, 9, 16]. A wide range of cognitive abilities has also been reported in WSS patients, with most exhibiting mild to moderate intellectual disability. Rare instances of WSS patients with normal cognition have been reported [4, 7]. Additional, less consistent features of WSS include anodontia, dysrhythmia, keratoconus, and syndactyly. Some who have undergone brain imaging have revealed white matter changes [4, 8–10, 14].

Interestingly, WSS has been described almost exclusively in consanguineous families (*n* = 27/29, 93.1%). Two nonconsanguineous families were described prior to the discovery of the gene associated with WSS; thus, molecular confirmation of this diagnosis has not been published [4]. WSS is caused by mutations in the gene encoding DCAF17 (DDB1 and CUL4 associated factor 17, C2ORF37), a transmembrane nuclear protein of uncertain function. Nine putative loss of function mutations in* DCAF17* have been reported in association with WSS [6, 9–13, 15]: three nonsense mutations [c.341C>A (p.S114X), c.387G>A (p.W129X), and c.906G>A (p.W302X)], four intronic mutations that are predicted to result in* DCAF17* missplicing (c.127+3delTAGinsAA, c.321+1G>A, c.1091+6T>G, and c.1422+5G>T), and two single nucleotide deletions (c.50delC and c.436delC). With the exception of c.436delC, a founder mutation identified in multiple families from the Arabian Peninsula [6], each of these is a private mutation found in a single consanguineous family. To our knowledge, no WSS patients with compound heterozygote* DCAF17* mutations have been reported. In this paper, we describe an additional consanguineous family with features of WSS. Homozygosity mapping using SNP array was used to identify* DCAF17* as a candidate gene.

## 2. Materials and Methods

Peripheral blood samples were obtained from Patients 1 and 2 with informed consent following the guidelines of the Institutional Review Board at the Johns Hopkins School of Medicine. Genomic DNA was extracted using the QIAamp DNA Blood Midi kit (Qiagen, Valencia, CA, USA) and SNP array was performed using the Illumina HumanOmni1-Quad (1 million markers; Illumina, San Diego, CA, USA). BeadChips were imaged using the Illumina Bead Array reader and allele ratios/signal intensities were analyzed with the CNV Partition 2.4.4.0 algorithm in KaryoStudio (v.1.4.3.0) and GenomeStudio (v.2010.3) (Illumina). The coding exons and flanking intronic regions of* DCAF17* were sequenced at Centogene (Rostock, Germany). All genomic coordinates are based on February 2009 Human Genome Build (hg19).

## 3. Case Presentations

### 3.1. Patient 1

Patient 1 (pedigree position V-3) presented with his family from Qatar on referral from a local pediatric endocrine colleague to our clinic at the age of 17-4/12 years for evaluation of a potential unifying diagnosis for his constellation of features including HH, cognitive delay, pectus carinatum, and microcephaly. History included an uncomplicated pregnancy and delivery. He had early delays, walking at 16–18 months and using his first words at 18 months. Formal developmental assessment at 6 years of age revealed no specific delays; yet his parents reported consistent global inability to keep up with peers. Though attending 11th grade at the time of clinical presentation, his parents estimate he is at least 4 grade levels behind age-matched peers. In terms of his physical features, patient 1 had thick, dark hair of normal texture until 6 years of age when it began to thin. Initially there was diffuse hair loss over the scalp, then localized to the bitemporofrontal region, as in male-pattern progressive alopecia. At 15 years of age, he was evaluated for lack of all sexual development. Laboratory evaluations at that time revealed low LH (0.51 *μ*IU/mL; normal 1.1–7.0), FSH (0.38 *μ*IU/mL; normal 1.7–12.0), and testosterone (0.27 ng/mL; normal 3.0–10.6), making the diagnosis of HH. After treatment with testosterone, the patient's voice deepened, he developed mild axillary and facial and pubic hair (Tanner IV), and there was mild genital development. He had delayed primary and secondary tooth eruption and now has multiple caries throughout. He has poor oral hygiene, reported to be similar to that of his brother, yet the latter does not have caries and neither has tooth breakage. On review, he has grossly normal hearing, no additional chronic medical conditions, and no motor movement abnormalities and he is able to smell. He wears glasses for mixed hyperopia and myopia with astigmatism. His growth has always been at low average for height and weight. On exam, weight is just above the 3rd percentile, height is 10th percentile, and his OFC is <−2 SD below the mean (50% for 7-year-old male). He appears to be microcephalic with a horizontal ridge on the superior forehead and bitemporal narrowing. The auricles (>2 SD) and nose are large. Chest circumference is 75th percentile with low, widely spaced nipples, and he has an asymmetric pectus carinatum with a bell-shaped inferior rib margin. He appears to be dolichostenomelic though arm span : height ratio is upper-normal at 1.05. Upper segment lower segment ratio is 1.05 (normal) and limb segments are proportionate with full range of motion of small, medium, and large joints throughout. Several keloid-type scars are present on the posterior right shoulder, back, and foot from a prior accident.

### 3.2. Patient 2

Patient 2 (V-2) presented at 19-0/12 years of age with her brother on referral from a local adult endocrinologist for evaluation of a potential genetic cause for her primary amenorrhea and gonadal dysgenesis. Her parents were first concerned about her lack of pubertal development at 15 years of age. Laboratory evaluations at that time revealed elevated FSH, LH and low estradiol (exact values unavailable), and a normal karyotype (46,XX). She was treated with hormone therapy (i.e., oral estrogens and progesterone; dose unknown) to induce secondary sexual characteristics and menses, now monthly. There was one low platelet value prior to presentation at our institution (111K). The patient denied bleeding problems, palpable bruising, or petechiae and none of these features were appreciated on exam. Pelvic ultrasound imaging showed hypoplastic ovaries and uterus. Her scalp hair began to thin at 11 years of age and she was recently treated with minoxidil, a topical vasodilator, to promote scalp hair growth. On review, her family reports her speech has become less clear over the last few months. She is otherwise healthy without other chronic medical conditions and she has always had normal, age-matched global development. On exam, her weight is 25–50th percentile and height is 50th percentile and she is normocephalic with sparse hair around the temporal region. She has no pectus and breast development is Tanner 3 with pubic hair at Tanner 3. Limb segments and limbs to trunk are proportionate with full range of motion of all joints and no camptodactyly.

**Figure 1:**
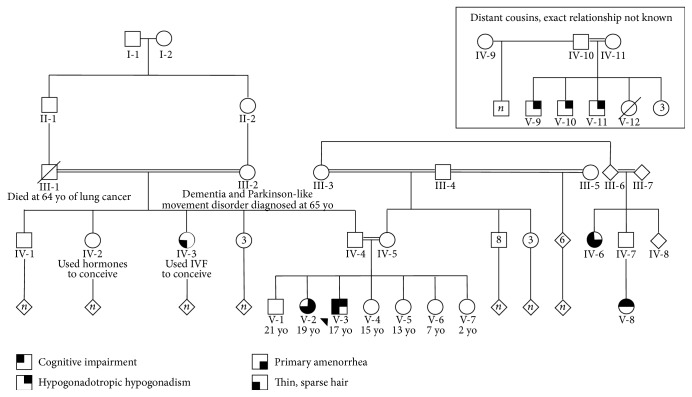
Pedigree illustrating multiple instances of consanguinity. Patient 1 (V-3), the proband, is indicated with an arrow. The proband's parents (IV-4 and IV-5) shared a common grandfather approximately 5 generations ago. Individual's clinical phenotypes are specified according to the key. The individuals in the inset are related to both the maternal and paternal lineages, but exact relationships are not known.

Family history for both patients is significant for multiple instances of consanguinity throughout the pedigree and several family members were significant for infertility (Figure 1). Patients 1 and 2 have a 21-year-old brother (V-1) and 4 sisters ranging in age from 2 to 15 years of age who are all healthy with normal pubertal development, hair, and cognition (V-4 to V-7). The parents of our patients entered puberty at 16 years of age and they are at average height (180 cm and 158 cm) with normal hair, cognitive development, and fertility. There are 2 paternal aunts (IV-2 and IV-3) who underwent infertility treatment (i.e., IVF, hormone treatment) to conceive additional pregnancies after initial successful spontaneous gestations; one paternal aunt (IV-3) also has a history of hair loss. The paternal grandmother (III-2) carries a diagnosis of dementia since 65 years of age, complicated by a Parkinson-like movement disorder; she conceived all her pregnancies naturally per available history. The paternal grandfather (III-1) died at 64 years of age from lung cancer though he was not a smoker. The mother of our patients (IV-5) is alive and well with 8 full brothers, 3 full sisters, and 6 paternal half-siblings. There is a maternal cousin once-removed from our patients (IV-6) who never entered puberty and has hair loss and cognitive delay and another maternal second cousin (V-8) without pubertal development and difficulty in school. There are several distant cousins (multiple siblings sharing the same mother and father, V-9 to V-11) related through both the maternal and paternal sides of our patients who are described to have a similar body shape as patient 1, did not develop secondary sexual characteristics, and were infertile. The father of one of these affected family branches (IV-10) remarried and had several subsequent children with normal pubertal development.

## 4. Results

**Figure 2:**
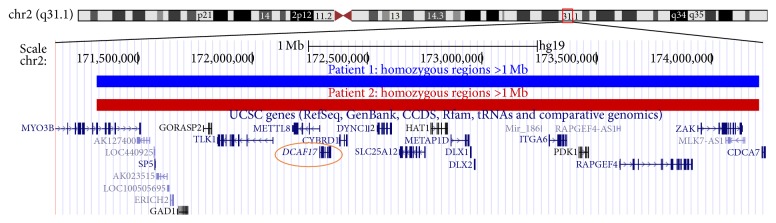
Illumina HumanOmni1-Quad SNP array indicates a shared region of homozygosity on chromosome 2q31.1. This region is approximately 2.9 Mb and contains* DCAF17*, the gene associated with WSS. This graphic was constructed using the UCSC Genome Browser (GRCh37/hg19).

Based on the highly suggestive autosomal recessive pedigree, we performed a SNP array on patients 1 and 2 to examine shared areas of homozygosity for genes associated with HH. The SNP arrays revealed a male genotype and female genotype with no significant copy number alterations for patients 1 and 2, respectively. However, as expected due to the consanguineous family history, in each individual there were multiple, large homozygous regions (>1 Mb in length) with normal copy number. These homozygous regions ranged in size from 1 Mb to 12.5 Mb and represented approximately 2.8% of the genome in each patient. Overall, the two siblings shared 10 homozygous regions totaling to 30.5 Mb (0.99% of genome). The Genomic Oligoarray and SNP array evaluation tool v3.0 (http://firefly.ccs.miami.edu/cgi-bin/ROH/ROH_analysis_tool.cgi) was used to search for genes associated with HH in the shared regions of homozygosity [17]. A search of OMIM Clinical Synopsis fields using the term “hypogonadism” identified* DCAF17* as the only candidate gene in the shared regions of homozygosity (Figure 2). Sequencing of the coding exons and flanking intronic regions of* DCAF17* in patient 1 revealed homozygosity for the previously described founder mutation (c.436delC; p.Ala147Hisfs^*∗*^9) [6]. Targeted* DCAF17* sequencing in patient 2 identified the same homozygous mutation. Monetary resources were not available to confirm the heterozygous carrier status of the parents. However, as noted, this family's* DCAF17* mutation was previously described, there was no evidence of a large* DCAF17* deletion, and our patients' phenotype was consistent with WSS.

## 5. Discussion

In this report, we have presented the clinical and molecular findings for two new WSS patients. Similar to most patients described in the literature, these patients are from a consanguineous family from the Middle East. Despite the identification of a Middle Eastern* DCAF17* founder mutation associated with WSS, we suggest that the presence of HH manifesting as delayed pubertal development in any consanguineous family should prompt consideration of this syndrome. There is a general paucity of WSS patients reported in the literature; therefore, the validity of this apparent geographic distribution cannot be determined. It is possible that the diagnosis of WSS is not considered in other populations because individuals do not have all the cardinal features of the condition (i.e., HH, diabetes mellitus, alopecia, and sensorineural hearing loss). Even in the family presented here, the phenotype is more variable among the affected individuals than presented in earlier articles. Overall, it is likely that there are more individuals with WSS in whom the diagnosis has not been considered and testing has not been pursued.

Although we have only been able to confirm the WSS diagnosis in patients 1 and 2 from this Qatari family, we suspect, based on the family history, that there are additional family members who are affected (Pedigree positions: IV-2, IV-3, IV-6, V-8, V-9, V-10, and V-11). Of those, 2 paternal aunts (IV-2 and IV-3) to patients 1 and 2 demonstrated infertility which was reportedly treated successfully with hormone supplementation and IVF, resulting in pregnancy. Unfortunately additional details of their clinical course are not available from these individuals and molecular testing has not been possible to validate that pregnancy is possible in WSS patients with HH. A survey of the literature revealed one female who potentially had WSS that may have been able to bear children [8]. Although molecular testing of this mother was not possible, she was thought to have WSS and was able to conceive and deliver four live-born offspring. It is unknown if assisted reproductive technologies were utilized to achieve these pregnancies (personal communication). This is an intriguing point for patients and families with WSS and the associated HH in that fertility may be possible for affected individuals. It should be noted that there is a wide range of cognitive abilities in affected individuals. Individuals with WSS with normal cognition would be able to raise children and therefore could benefit from a variety of reproductive technologies including sperm or egg retrieval, ICSI, IVF, and/or surrogacy if uterine or gonadal development was insufficient. Clinicians are encouraged to report such events in patients confirmed to have WSS to allow other patients and their healthcare providers to benefit from this experience.

This study further illustrates the utility of SNP array in consanguineous families to efficiently and inexpensively direct further study of shared genomic regions for disease-causing gene variants among affected individuals [18]. Even with the upsurge of whole exome sequencing (WES), SNP array still remains a cost effective method of mapping homozygous regions in consanguineous families, particularly those with distinct phenotypes [19]. Despite the falling price of WES, it is still costly to perform WES for multiple individuals in a single family. High resolution SNP array can be used to determine regions containing potential candidate genes in consanguineous families and can be followed by WES in a single individual or Sanger sequencing of candidate genes in multiple affected individuals. In addition, homozygosity mapping using high resolution SNP array can assess regions of the genome that are not represented in WES data including noncoding regions or those not covered due to technical limitations including low read depth or overlap with paralogous sequences [18]. In this study, cost and the high likelihood of finding a small number of candidate genes that could be followed up with Sanger sequencing were both considerations when choosing the SNP array-first testing strategy. Homozygosity mapping using SNP array is not without limitations, however. The resolution of homozygous regions that can be detected by SNP array is directly proportional to the number of SNPs on the array. Thus, use of a high resolution array is particularly important in consanguineous families manifesting phenotypes with high locus heterogeneity to limit the number of candidate genes that require follow-up testing.

In summary, this family illustrates the variable phenotype associated with WSS and suggests that this diagnosis should be considered in any consanguineous family presenting with HH manifesting as delayed pubertal development, alopecia, intellectual compromise, and/or extrapyramidal movements.

## Abbreviations

HH: Hypogonadotropic hypogonadism
WES: Whole exome sequencing
WSS: Woodhouse-Sakati syndrome.
